# Recruitment and retention of health professionals in the European Union: lessons from country experiences

**DOI:** 10.1186/1472-6963-14-S2-O20

**Published:** 2014-07-07

**Authors:** Gilles Dussault

**Affiliations:** 1 Instituto de Higiene e Medicina Tropical, Lisbon, Portugal

## 

Following up on J Buchan’s presentation (Right time right place? New evidence on effective health workforce distribution and retention), This presentation will focus on lessons derived from a literature review of studies on interventions to address recruitment and retention of health professionals in the European Union. Most, if not all, EU countries experience difficulties in recruiting in some professions (nursing, midwifery) and in some areas of health services (mental health, home care, emergency services), and in some geographical areas (remote, isolated, poor). Countries also have problems in retaining professionals tempted to migrate to other countries or to other sector of activities, or in attracting back those who have left, such as older nurses or physicians who have retired but who may still be interested in practicing. Few countries have developed policies to address these problems, but some have made systematic efforts in that direction. The presentation will describe some examples and discuss their effectiveness and the lessons which other countries can derive from them.

